# EMP1 as a Potential Biomarker in Liver Fibrosis: A Bioinformatics Analysis

**DOI:** 10.1155/2023/2479192

**Published:** 2023-03-22

**Authors:** Xuchen Chen, Xinliang Lv, Manman Han, Yexiao Hu, Wanqiong Zheng, Haibo Xue, Zhuokai Li, Kui Li, Wei Tan

**Affiliations:** ^1^Department of General Surgery, Wenzhou Hospital of Integrated Traditional Chinese and Western Medicine Wenzhou, Zhejiang, China; ^2^Department of Hepatobiliary and Pancreatic Surgery, Lishui Municipal Central Hospital, The Fifth Affiliated Hospital of Wenzhou Medical University Lishui, Zhejiang, China; ^3^Department of Gastroenterology, The First Affiliated Hospital of Wenzhou Medical University Wenzhou, Zhejiang, China; ^4^Department of Blood Transfusion, Lishui Central Hospital, Fifth Affiliated Hospital of Wenzhou Medical College Lishui, Zhejiang, China

## Abstract

Liver fibrosis is a wound-healing response to chronic injury, which may result in cirrhosis and liver failure. Studies have been carried on the mechanisms and pathogenesis of liver fibrosis. However, the potential cell-specific expressed marker genes involved in fibrotic processes remain unknown. In this study, we combined a publicly accessible single-cell transcriptome of human liver with microarray datasets to evaluate the cell-specific expression patterns of differentially expressed genes in the liver. We noticed that *EMP1* (epithelial membrane protein 1) is significantly active not only in CCl_4_ (carbon tetrachloride)-treated mouse liver fibrosis but also in BDL (bile duct ligation)-induced liver fibrosis and even in human fibrotic liver tissues such as alcoholic hepatitis, NASH (nonalcoholic steatohepatitis), and advanced stage liver fibrosis. Furthermore, we demonstrated that EMP1 is a specific fibrotic gene expressed in HSCs (hepatic stellate cells) and endothelial cells using the Protein Atlas single-cell transcriptome RNA-sequencing clustering. Its expression was significantly elevated in fibrotic HSCs or CCl_4_ and NASH-induced fibroblasts. Previous research revealed that *EMP1* plays a role in proliferation, migration, metastasis, and tumorigeneses in different cancers via a variety of mechanisms. Because HSC activation and proliferation are two important steps following liver injury, it would be interesting to investigate the role of EMP1 in these processes. All of this information suggested that EMP1 could be used as a novel fibrotic liver marker and a possible target in the future.

## 1. Introduction

Liver fibrosis is an abnormal accumulation of extracellular matrix (ECM) proteins, such as collagen, that occurs in the majority of chronic liver disease, and cirrhosis, liver failure, and portal hypertension are all symptoms of advanced liver fibrosis [1]. Liver inflammation is caused by chronic liver damage that disrupts the physiological architecture of the liver tissue [2]. During the injury, hepatocytes apoptosis and emit damage-associated patterns, which recruit and activate lymphocytes and macrophages, as well as promote pro-fibrotic myofibroblast activation [3]. These myofibroblasts are primarily derived from transdifferentiation resident hepatic stellate cells (HSCs) [4]. However, the fate of liver might either be an anti-fibrotic, scar-dissolving stage, or an unconstrained fibrosis-promoting stage by liver non-parenchymal cells [5]. A number of pathways and mediators, such as autophagy, endoplasmic reticulum stress, oxidative stress, retinol and cholesterol metabolism, epigenetics, and receptor-mediated signals, demonstrate the complexities of HSC activation [6, 7]. The novel possible marker genes that may be important in these processes, on the other hand, remain unexplored.

In this study, we combined single-cell RNA sequence data from liver samples with a conventional microarray dataset from public database to identify marker genes that are expressed in HSCs and respond during the fibrotic process. We discovered that *EMP1* (epithelial membrane protein 1) is significantly activated not only in CCl_4_ (carbon tetrachloride)-treated mice liver, but also in BDL (bile duct ligation)-induced mice liver fibrosis and even in human fibrotic liver tissues such as alcoholic hepatitis, NASH (nonalcoholic steatohepatitis), and also liver with advanced stage disease. Furthermore, we proved *EMP1* is a particular fibrotic gene expressed in HSCs and endothelial cells (EC) utilizing the single-cell transcriptome RNA-sequencing clustering result from ProtinAtlas database, and its expression was dramatically elevated in both CCl_4_ and NASH-induced fibroblasts. Previous studies found that *EMP1* regulates cancer cell migration and proliferation, and we suspect it has a similar role in activated HSCs [8, 9]. The epithelial membrane proteins (EMPs) are encoded by the peripheral myelin protein 22 kDa (PMP22) gene family, and involved in tumor cell migration, growth, and differentiation. EMP1 is also known as CL-40, tumor-associated membrane protein. The EMP1 protein consists of 157 amino acids and is a glycoprotein containing four highly conserved hydrophobic transmembrane domains localized on the membrane. All of this information showed that EMP1 might be employed as a novel fibrotic liver marker and could be a potential target in the future.

## 2. Materials and Methods

### 2.1. Gene Expression Profile Data

All gene expression microarray matrix were collected from the NCBI (National Center for Biotechnology Information) GEO (Gene Expression Omnibus), which is a database repository of high throughput gene expression data and hybridization arrays, chips, and microarrays [10].

### 2.2. Differentially Expressed Gene (DEG) Analysis

The expression matrix from microarray dataset GSE141821 [11], GSE55747, and GSE80601 [12] downloaded from GEO was automatically loaded into R statistical software (version 3.8.0, https://www.r-project.org/) and DEGs was generated using the limma package (version 3.46.0) [13]. Genes are considered to be differentially expressed if they have an absolute log2 fold change of >1 at FDR of <0.05. For all the other microarray dataset including GSE103580 [14], GSE139994 [15], GSE27640 [16], GSE28619 [17], GSE40041 [18], GSE49541 [19], and GSE73499 [20], the GEO2R analysis was used to acquire EMP1 expression values and construct differential expressions [21]. All the datasets related information could be found in the GEO database. The dataset information is shown in Table 1.

### 2.3. Function Enrichment Analysis

We uploaded the DEGs into Metascape (https://metascape.org/) and conducted Gene Ontology (GO), Kyoto Encyclopedia of Genes and Genomes (KEGG) enrichment analyses, and Minimal Common Oncology Data Elements (MCODE) enrichment analysis [22]. The results of GO and KEGG analysis were visualized in the R ggplot2 package (v 3.3.3). Meanwhile, DEGs were submitted into the STRING (Search Tool for the Retrieval of Interacting Genes/Proteins) database (v11.0) using the medium confidence (interaction score >0.400) parameter [23]. Functional annotation enrichment was also performed in STRING and top enriched pathways were visualized in R using ggplot2 package (v 3.3.3).

### 2.4. Liver Tissue Single-Cell Analysis

Because numerous cells are involved in the course of liver fibrosis, the expression of common genes in these cells was examined to see if they induce fibrosis by controlling the function (migration) of these cells. In this study, we used the Protein Atlas [24] and PanglaoDB databases [25] to investigate for specific expression patterns of *EMP1*. Protein Atlas is an interactive open-access database (http://www.proteinatlas.org/) to allow genome-wide exploration of the impact of individual proteins on clinical outcomes. PanglaoDB (https://panglaodb.se/) is a database for the scientific community interested in investigating single-cell RNA-sequencing research from mice and humans.

### 2.5. GEPIA Database Analysis

The GEPIA (Gene Expression Profiling Interactive Analysis) [26] is a website that contains the sequenced RNA expression data of 9736 cancers and 8587 normal samples from the TCGA and GTEx (the genotype-tissue expression) projects (http://gepia.cancer-pku.cn). Pearson's correlation was utilized to validate the strong positive correlations of EMP1 with other fibrotic-related genes, as well as the expression matrix of normal liver tissues from GTEx [27].

## 3. Results

### 3.1. Fibrotic-Related Gene Expression Patterns in CCl_4_-Induced Mouse Liver Samples

**Figure 1:**
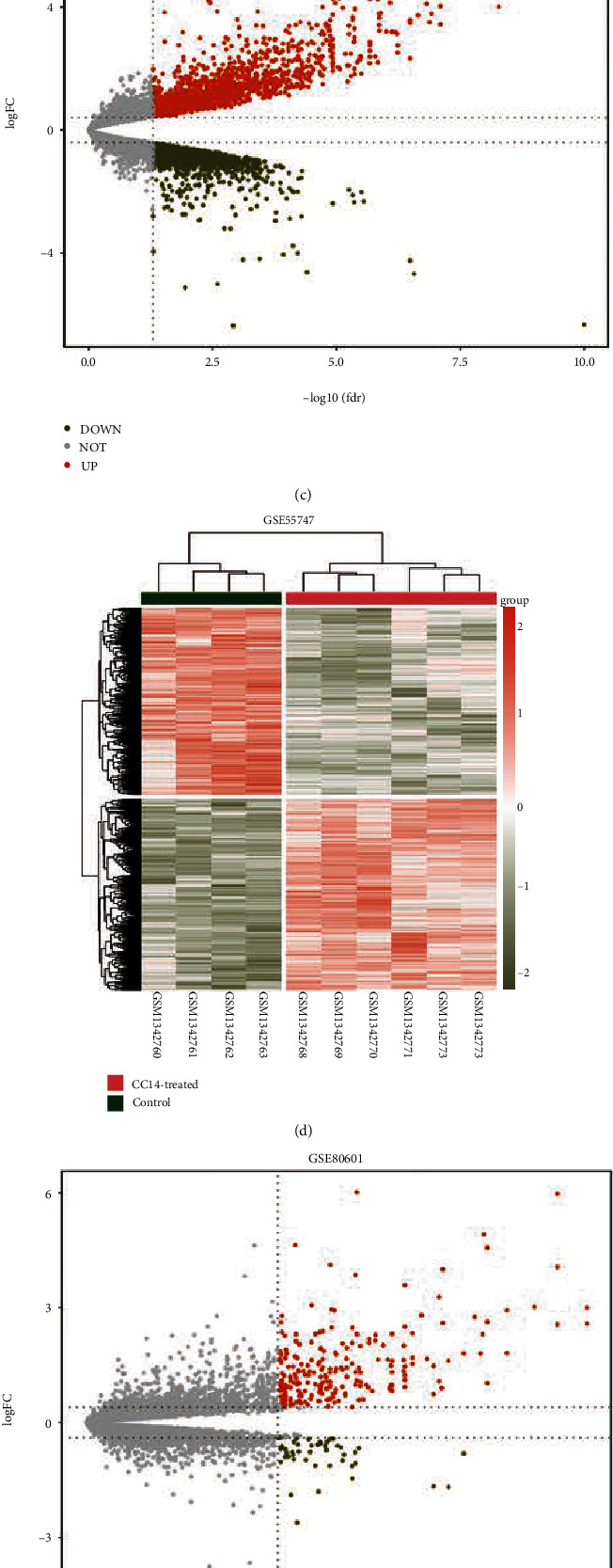
Identification of DEGs in CCl_4_-treated mouse liver samples. DEGs were obtained by comparing untreated and CCl4-induced fibrotic mouse liver tissues from three GEO datasets: GSE14182, GSE55747, and GSE80601. (a)–(c)) Volcano plot of the distribution of all DEGs between untreated control and CCl_4_-treated liver samples from GSE14182, GSE55747, and GSE80601, respectively, with a threshold of |log_2_(foldchange)| >1 and adjusted *P* < 0.05 (the brick red, upregulated DEGs; grass green: downregulated DEGs; grey: unchanged). (d)–(f) The heatmap plot of DEGs across the samples (CCl_4_-treated vs Control) from GSE14182, GSE55747, and GSE80601, respectively. Colors above the heatmap indicate sample groups. Each row of the heatmap represents the normalized expression values of one differentially expressed gene across all samples (grass green, low expression; brick red, high expression). (g) and (h) Venn diagram of DEGs from 3 different microarray dataset. CCl_4_, carbon tetrachloride; DEG, differential expressed genes; GEO, Gene Expression Omnibus.

To investigate the mechanisms of liver fibrosis, we analyzed the DEGs in three GEO datasets between CCl_**4**_-induced fibrotic and control samples. In GSE141821, 45 upregulated and 7 downregulated DEGs were found (|log2 (foldchange)| >1, adjusted *P* = 0.05; Figure 1(a) and 1(b)). On the other hand, Figure 1(c) and 1(d) demonstrate that in GSE55747 fibrotic mouse liver samples, there were 1817 upregulated and 1778 downregulated DEGs with a |log2 (foldchange)| > 1 and adjusted *P* < 0.05. In GSE80601, following analysis, 1778 downregulated and 1817 upregulated DEGs were found in CCl4-induced fibrotic liver tissues (|log2 (foldchange)| > 1, adjusted *P* < 0.05; Figures 1(e) and 1(f)). We identified 62 upregulated genes and 5 downregulated genes by intersecting the DEGs from three datasets (Figures 1(g) and 1(h)). These genes encode a number of ECM proteins, including *Col3a1*, *Col4a1*, and *Col1a1*. In the meanwhile, genes encoding ECM degrading enzymes including *Timp2*, *Mmp2*, and *Adamts5* were considerably upregulated. Acute-phase protein expression of *Saa3* (serum amyloid A3) significantly elevates in response to acute and chronic inflammatory stimuli. This finding highlights the fact that the ECM is altered after CCl_4_ treatment. Also, *Cd14*, *Cd53*, and *Cd68*, which are markers for monocytes and macrophages, were considerably increased. It implies an increase of macrophage contents and increased immune filtration level following the injury. Cytochrome P450 gene family members, such as *Cyp7b1* and *Cyp2d40* are among downregulated genes. In addition, after the injury, *Hsd3b5* (hydroxy-delta-5-steroid dehydrogenase, 3 beta- and steroid delta-isomerase 5) was down regulated. The function of normal liver cells is impacted by liver injury. The upregulated and downregulated DEGs were shown using Venn diagrams (Figures 1(g) and 1(h), respectively).

**Figure 2:**
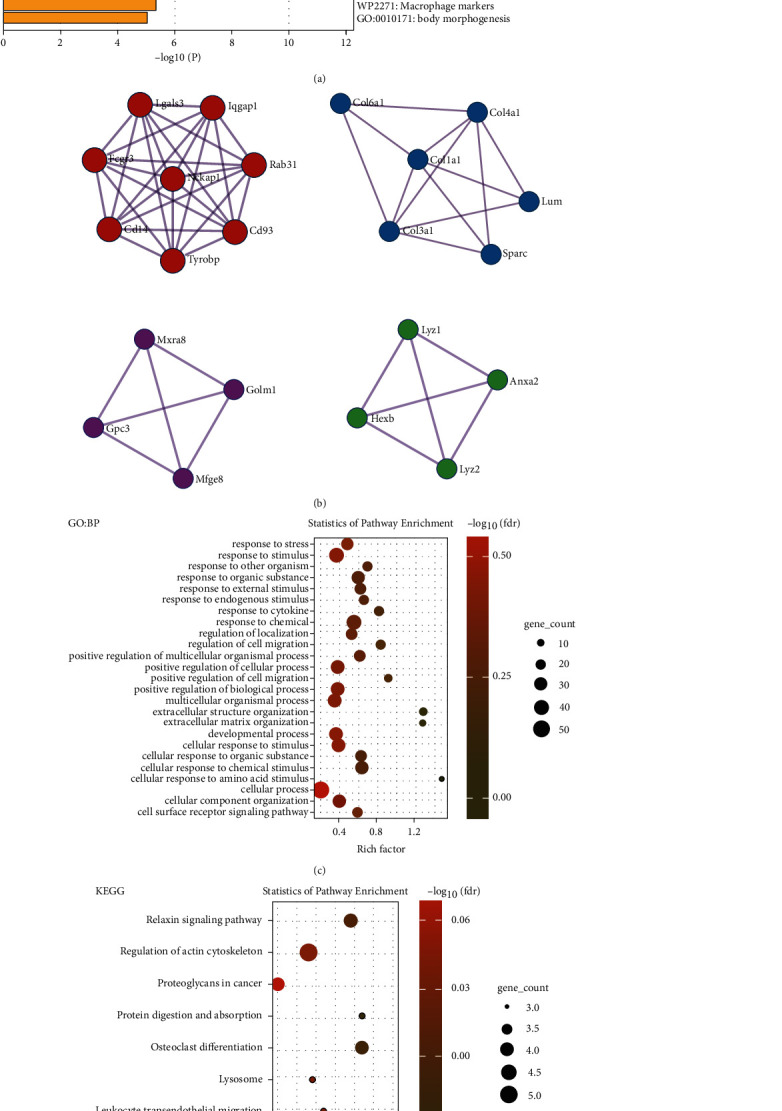
Functional enrichment analysis of DEGs. (a) Bar chart of clustered enrichment ontology categories (including both GO and KEGG terms) by Metascape (colored by *P*-values; minimum overlap: 3; *P* < 0.01; minimum enrichment: 1.5). (b) MCODE enrichment analysis of PPI network by Metascape. Functional enrichment analysis of GO: BP (c) and KEGG (d) using PPI generated by STRING. DEGs were imported into the STRING database to generate a PPI network with a 0.4 minimum necessary interaction score. GO, Gene Ontology; KEGG, Kyoto Encyclopedia of Genes and Genomes; MCODE, Molecular Complex Detection; PPI: protein–protein interaction.

The enrichment analysis was performed by Metascape (Figure 2(a)). Pathways involved includes ECM organization (GO: 0030198), positive regulation of cell migration (GO: 0030335), collagen degradation (R-MMU-1442490), and positive regulation of response to external stimuli (GO: 0032103).  Figure 2(b) depicts the MCODE analysis for PPI (protein–protein interaction) models by Metascape. The ECM protein cluster is part of the core PPI interaction network (*Col1a1*, *Col3a1*, *Col4a1*, *Col6a1*, *Lum*, and *Sparc*; Figure 2(b)). Furthermore, we used STRING for functional annotation enrichment, and the top enriched pathways were displayed in R using the ggplot2 tool (v 3.3.3). GO:BP enrichment indicated that pathways, such as stress response and cytokine are altered (Figure 2(c)), whilst KEGG enrichment indicated an alteration in actin cytoskeleton regulation and leukocyte transendothelial activation (Figure 2(d)). These enrichment pathway findings are mostly consistent with the fibrosis process in the liver.

### 3.2. EMP1 Is a Marker Gene for CCl_4_-Treated Liver Samples

**Figure 3:**
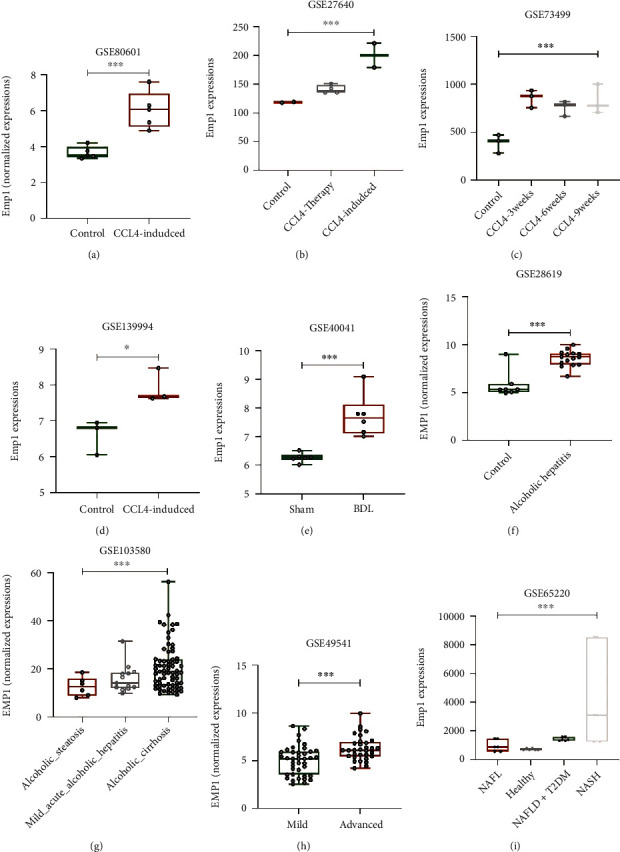
*EMP1* is a marker gene for fibrotic liver in both animal and human samples. *Emp1* upregulation in CCl_4_-treated mouse liver samples compared with normal tissues in GSE80601 (a), GSE27640 (b), GSE73499 (c), and GSE139994 (d). *Emp1* expression elevation in mice BDL model compared with sham group (e). Positive association of *EMP1* expression with alcoholic hepatitis (GSE28619) (f) and alcoholic cirrhosis (GSE103580) samples (g). Increased expression of *EMP1* in liver samples with advanced stage of fibrosis (stage 3–4) compared with mild (stage 0–1) (h). In the rat model, *Emp1* expression is greatest in the NASH group compared to the NAFL, NAFLD + T2DM, and healthy control groups. BDL, bile duct ligation (i); CCl_4_, carbon tetrachloride; EMP1, epithelial membrane protein 1; NAFL, nonalcoholic fatty liver; NAFLD, nonalcoholic fatty liver disease; NASH, nonalcoholic steatohepatitis; T2DM, type 2 diabetes; ∗*P* < 0.05  and ∗∗∗*P* < 0.01.

We noticed that CCl_4_ treatment caused a rapid increase in *Emp1* expression in mouse liver tissues from various datasets (Figures 3(a)–3(d)). The GSE80601 expression profiles of liver tissues treated with CCl_4_ (*n* = 6) and control liver tissues (*n* = 5) treated with oil for both 6 weeks from Balb/c mice revealed that *Emp1* expression significantly increased following CCl_4_ treatment (log_2_FC = 2.461, *P* = 2.39 × 10^−4^; Figure 3(a)). In GSE27640 after 18 weeks of CCl_4_ treatment in the mouse model, the *Emp1* gene was significantly upregulated (log_2_FC = 1.673, *P* = 2.61 × 10^−3^) compared to normal control liver tissues. Erlotinib, an EGF receptor inhibitor that can reduce liver fibrosis and the development of hepatocellular carcinoma, suppressed the increased expression of *Emp1* in GSE27640 (Figure 3(b)). Meanwhile, in GSE73499 following 3, 6, and 9 weeks of CCl_4_ administration, the expression of the *Emp1* gene was also significantly upregulated in the rat liver cirrhosis model (log_2_FC = 1.117, *P* = 6.13 × 10^−3^) (Figure 3(c)). When compared to samples of rat livers treated with a control vehicle, the expression of Emp1 in the liver tissue of rats treated with CCl_4_ was significantly upregulated (log_2_FC = 1.416, *P* = 0.035) in GSE139994. Taken together, these findings suggested that the liver tissues response to CCl_4_-induced acute and chronic injury triggers a rapid increase in *Emp1* in mouse model.

### 3.3. EMP1 Is a Marker Gene for Fibrotic Liver Samples

Aside from the CCl_4_-treatment model, similar findings have been made in other liver fibrosis models, including human clinical fibrotic liver samples caused by conditions like NAFLD (nonalcoholic fatty liver [NAFL] disease). For instance, when compared to the control group, *Emp1* is rapidly activated in GSE40041 in acute or chronic damage mouse models by sham or BDL for either 48 hours or 28 days, respectively (log_2_FC = 1.152, *P* = 3.26 × 10^−4^; Figure 3(e)). According to the GSE28619 dataset, hepatic gene expression profiling was examined by microarray in patients with alcoholism (*n* = 15) and normal livers (*n* = 7), and *EMP1* is considerably higher in alcoholic hepatitis tissues (GSE28619; log_2_FC = 2.73, *P* = 1.08 × 10^−5^; Figure 3(f)). *EMP1* is also substantially more highly expressed in alcoholic steatosis and cirrhosis than in moderate acute alcoholic hepatitis, according to the set of data (GSE103580; (log_2_FC = 0.325, *P* = 0.032; Figure 3(g)). Moreover, *EMP1* is greater higher expressed in samples of NAFLD liver biopsy tissues recovered from advanced (fibrotic stage 3–4) compared to moderate samples (fibrotic stage 0–1) in GSE49541 (log_2_FC = 1.152, *P* = 3.26 × 10^−4^; Figure 3(h)). Furthermore, in the different stages of rat fibrotic liver from GSE65220, Emp1 has the highest expression in NASH compared to other stages, such as healthy, NAFL, and NAFLD with T2DM (type 2 diabetes; log_2_FC = 2.157, *P* = 9.92 × 10^−3^; Figure 3(i)). All of this evidence suggests that EMP1 expression increased with the severity of the fibrotic liver malignancy and that it may play a role in the regulation of the liver injury response.

### 3.4. Dominant Expression of EMP1 in HSCs from Liver Single-Cell Transcriptome

**Figure 4:**
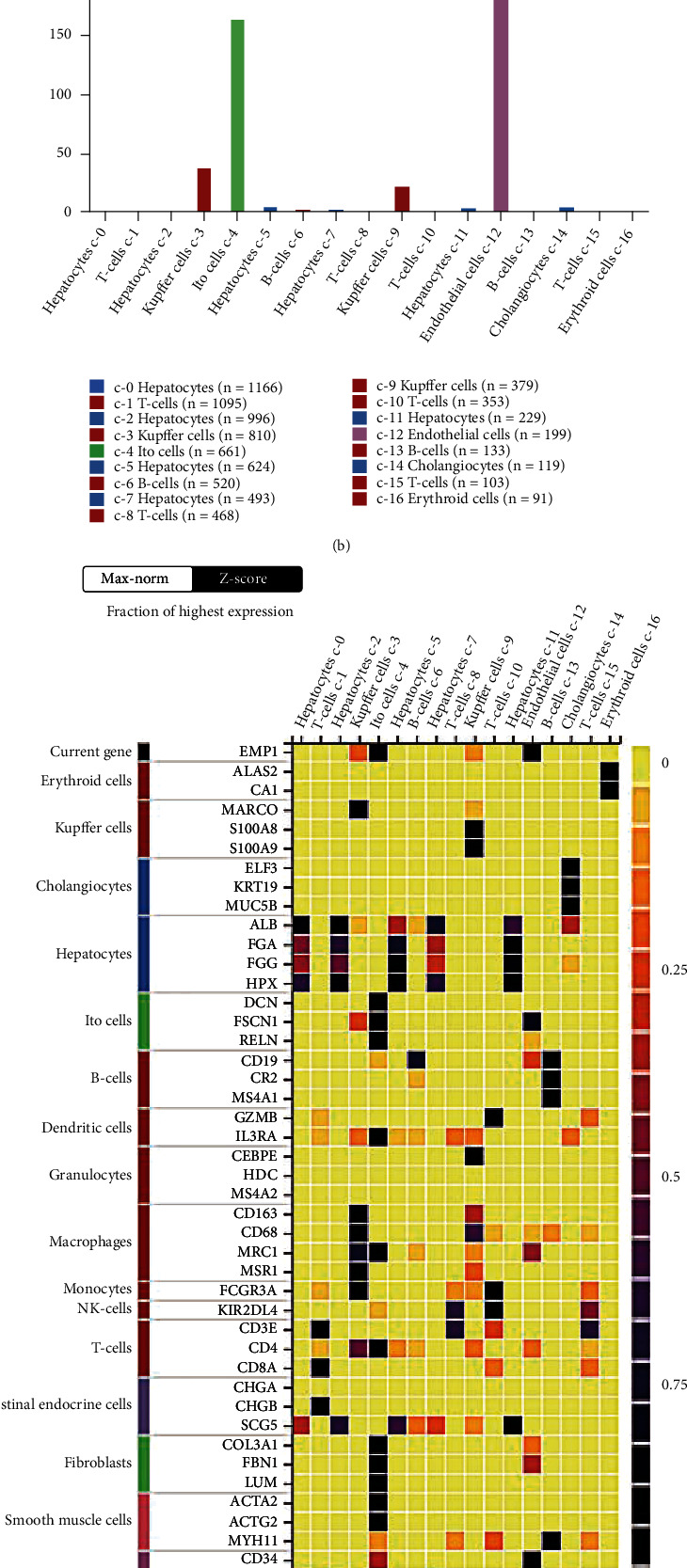
*EMP1* is expressed in activated fibrotic hepatic stellate. The cell type atlas given by Protein Atlas (https://www.proteinatlas.org/) validated the expression of *EMP1* in liver tissues. (a) Feature plot of *EMP1* overlaid with the UMAP projection of liver samples. (b) Bar plot showing the TPM expression of *EMP1* across different cell type clusters including hepatocytes, Ito cells, T cells, Kupfer cells, B cells, and others. (c) Heatmap plot of normalized *EMP1* expressions along with other marker genes across the different cell types in liver tissues. Significant positive association of *EMP1* expression with fibrotic HSCs (GSE120281) (d) and activated fibroblast introduced by either CCl_4_ treatment or Western diet-fed NASH model (GSE134512) samples (e). CCl_4_, carbon tetrachloride; EMP1, epithelial membrane protein 1; NASH, nonalcoholic steatohepatitis; TPM, transcripts per million; UMAP, Uniform Manifold Approximation and Projection. *P* < 0.05  and ∗∗∗*P* < 0.01.

Using The Human Protein Atlas (https://www.proteinatlas.org/), we investigated the expression profiles of different cell types in liver tissues to determine which cell type expresses EMP1 the most. We discovered that *EMP1* is exclusively expressed in EC (purple c-12 cluster) as well as Ito (perisinusoidal fat-storing cells, also known as stellate cells) of the liver as mesenchymal cells (grass green c-4 cluster) in the single-cell transcriptome clustering results for normal liver tissues. The data was displayed in a UMAP (Uniform Manifold Approximation and Projection plot; Figure 4(a)). According to the clustering results, *EMP1* expression is lower in Kupffer cells (brick red c-3 and c-6 clusters) and other cell types including immune cells and hepatocytes (c-2; Figure 4(a)). Ito cells had an average expression value TPM (transcripts per kilobase) of 161.3, while EC had an average TPM of 181.9, and *EMP1* expression is low in all other cell types (Figure 4(b)). The heatmap depicts the highest levels of EMP1 gene expression as well as other marker genes from various cell types, such as *CD34* for EC, *CD3E* for T cells, and *CD163* for macrophages. *EMP1* and *FSCN1* (Fascin actin-bundling protein 1) have the most similar expression patterns according to the dataset (Figure 4(c)).

### 3.5. EMP1 Expression Elevated in Activated HSCs and Liver Fibroblast

Since we know that the activation of HSCs into proliferative, fibrogenic myofibroblasts has long been recognized as the primary cause of hepatic fibrosis in both experimental and human liver injury. GSE120281, an mRNA profile of quiescent (Q) or fibrotic (F) HSC and liver sinusoidal EC (SEC) generated by RNA sequencing in control or CCl_4_-treated mice, was investigated. In fibrotic HSCs, compared to quiescent HSCs, *Emp1* expression is markedly increased in response to CCl_4_ treatment (log_2_FC = 4.895, *P* = 0.0008; Figure 4(d)). GSE134512 dataset that analyzed the transcriptome of activated fibroblasts from NASH livers and discovered that *Emp1* was significantly elevated in both CCl_4_-induced and NASH-induced fibroblasts when compared to activated HSCs (log_2_FC = 2.116, *P* = 0.00228; Figure 4(e)).

### 3.6. EMP1 Expression Correlated with ECM Proteins

**Figure 5:**
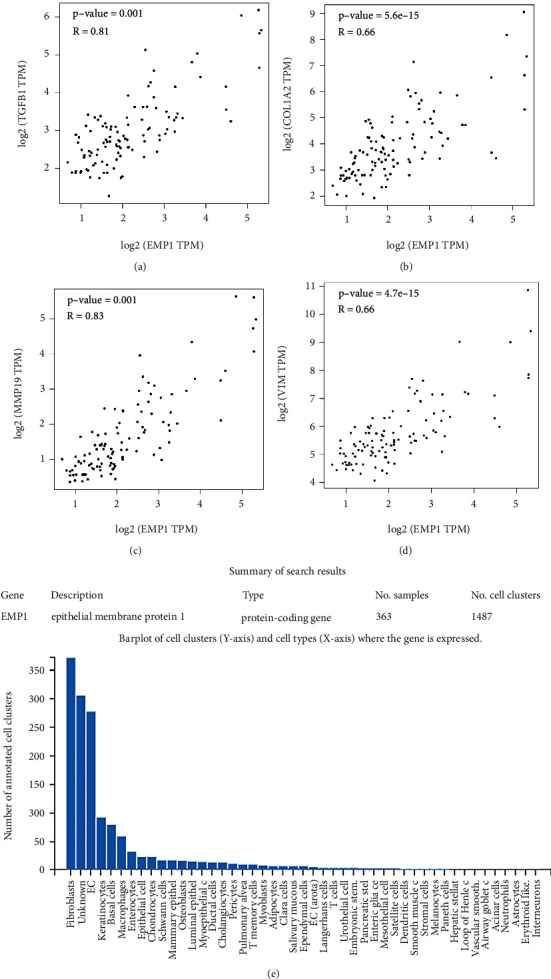
EMP1 expression positively correlated with fibrotic-related genes in liver. Significant positive correlations of *EMP1* expression with fibrotic-related genes including *TGFB1* (a), *COL1A2* (b), *MMP9* (c), and *VIM* (d), according to the expression profile from GTEx normal liver RNA-sequencing matrix in GEPIA2. (e) Barplot of cell clusters (*y*-axis) and cell types (*x*-axis) where the gene is expressed. Expression enrichment of *EMP1* in different cell types from 363 scRNA-seq samples and 1487 cell clusters according to PanglaoDB. COL1A2, collagen type *i* alpha 2 chain; EMP1, epithelial membrane protein 1; GEPIA2, Gene Expression Profiling Interactive Analysis; GTEx, Genotype-Tissue Expression; MMP9, matrix metallopeptidase 9; scRNA-seq, single-cell RNA sequencing; TGFB1, transforming growth factor beta 1; VIM, vimentin.

GEPIA was used to confirm the significantly positive association of *EMP1* expression with *COL1A2* (collagen type I alpha 2 chain), *TGFB1* (transforming growth factor beta 1), *MMP19* (matrix metallopeptidase 19), and *VIM* (vimentin) in normal liver samples (*r* > 0.60, *P* < 0.05; Figure 5(a)–5(d)). Then, we double-checked our findings using the PanglaoDB database. *EMP1* was shown to be highly expressed in fibroblasts, EC, followed by keratinocytes, but not in HSCs (Figure 5(e)). These results indicate that EMP1 may assist in the fibrotic progression after activation and transdifferentiation of HSCs in the development of liver fibrosis, therefore increasing liver fibrosis.

## 4. Discussion

The fundamental cause of liver fibrosis is the activation of HSCs, which then transform from quiescent, vitamin-A-storing cells into proliferative, fibrogenic myofibroblasts [28]. Deactivation of HSCs by Tcf21 (transcription factor 21), has been proved to suppress hepatic fibrosis progression in mice [29]. However, HSCs activation is a rather complex process that has yet to be fully understood.

In this work, we first demonstrated that EMP1 is one of the most elevated genes after CCl_4_-induced liver damage in mouse model. Then, we discovered that *EMP1* levels are also elevated in various liver samples after injury, such as the BDL model and alcoholic hepatitis in humans. EMP1 is expressed in fibroblasts and EC, according to single-cell transcriptome study of liver tissues. Furthermore, we observed that EMP1 expression was much greater in activated fibrogenic fibroblasts and was associated with typical fibrotic genes. The EMPs, which includes *EMP1*, *EMP2*, and *EMP3* are encoded by the growth arrest-specific 3 (GAS3)/peripheral myelin protein 22 kDa (PMP22) gene family [9]. The EMPs family has four putative transmembrane domains with about 160 amino acid residues [30]. Although the genes in this family are commonly implicated in cancer cell migration, proliferation, and differentiation, few studies have demonstrated their role in fibrosis. He et al. previously discovered that abnormal upregulation of *PMP22* in TGF-*β*-activated HSCs and CCl_4_-induced hepatic fibrosis model in mice, as well as the pro-fibrotic role of *PMP22* through aggravating TGF-*β*-induced HSC activation [31]. It would be interesting to look into and compare the role of the entire gene family in fibrotic processes in the liver.

Extensive research has been conducted into the role of EMP1 in pathogenesis and tumorigenesis of various cancer. In breast cancer, for example, EMP1 functions as a new marker for lobular and ductal invasive breast carcinoma differentiation, as well as a putative link with breast cancer invasion promotion [32]. With increasing histologic grade, the expression of EMP1, EMP2, and EMP3 decreases in the epithelial component and increases in the stromal component of phyllodes tumors [33]. In prostate cancer, *EMP1* is highly expressed in patients with a higher Gleason score, and increasing EMP1 levels significantly increases cancer cell migration, resulting in tumor metastasis, implying that EMP1 may play an important role as a positive regulator of tumor metastasis [8]. In ovarian cancer, *EMP1* was discovered to play a critical role as a negative regulator in ovarian serous tumors, and decreased EMP1 expression in serous tumors associated with increased disease severity [34]. EMP1 is also a biomarker of gefitinib resistance, and it has been linked to a lack of complete or partial response to gefitinib in lung cancer patient samples, as well as clinical progression to secondary gefitinib resistance [35]. In acute lymphoblastic leukemia, *EMP1* is a novel poor prognostic factor in pediatric leukemia that regulates prednisolone resistance, cell proliferation, migration, and adhesion [36]. In glioma, EMP1 regulates the cell proliferation, migration, and stemness through PI3K-AKT signaling and CD44 [37]. Given that the majority of research agree that EMP1 can enhance tumor growth, invasion, and migration, we hypothesize that EMP1 could also promote activated HSC proliferation and migration following injury. Stellate cell activation is characterized by proliferation and migration [38], and reducing proliferation may modify hepatic fibrosis, according to many published results [39–42]. However, further validating tests are required in the future to validate the hypothesis.

In our enriched analysis, the pathways involved includes ECM organization, positive regulation of cell migration, collagen degradation, and positive regulation of response to external stimuli. The enrichment analysis indicated that pathways such as stress response and cytokine are altered, whilst KEGG enrichment indicated an alteration in actin cytoskeleton regulation and leukocyte transendothelial activation. These enrichment pathway findings are mostly consistent with the fibrosis process in the liver.

There were several limitations in the present study. First, the included datasets were based on mice; we lack validation in human samples. Second, we did not perform the analysis to study the expression of EMP1 in different subgroups by different clinical factors such as sex and age. Third, lack of experiments to verify, and our analysis was performed using data sets from public databases but was not validated in larger data sets.

Taken together, these bioinformatic data imply that EMP1 might be used as a marker gene during liver fibrosis after injury and may have a potential role in HSCs, however additional validating experiments is needed in the future.

## Figures and Tables

**Table 1:** The characteristics of the included datasets.

Datasets	Species	Samples
GSE141821	Mice	*N* = 36, 18 CCl4 induced liver injury and 18 control
GSE55747	Mice	*N* = 17, 13 CCl4 induced liver fibrosis and 18 control
GSE80601	Mice	*N* = 10, 5 CCl4 induced liver fibrosis and 5 control
GSE103580	Human	*N* = 86, 67 cirrhosis samples and 19 control
GSE139994	Rat	*N* = 9, 6 CCl4 induced liver fibrosis and 3 control
GSE27640	Mice	*N* = 4, 2 CCl4 induced liver fibrosis and 2 control
GSE28619	Human	*N* = 22, 15 alcoholic hepatitis and 7 control
GSE40041	Mice	*N* = 24, 12 CCl4 induced liver fibrosis and 12 control
GSE73499	Rat	*N* = 12, 12 rat model of liver cirrhosis

## Data Availability

The simulation experiment data supporting this research article are available from the corresponding author or first author on reasonable request.
